# Gene-Edited Meat: Disentangling Consumers' Attitudes and Potential Purchase Behavior

**DOI:** 10.3389/fnut.2022.856491

**Published:** 2022-04-05

**Authors:** Daniel Martin-Collado, Tim J. Byrne, Jonh J. Crowley, Tom Kirk, Guillermo Ripoll, C. B. A. Whitelaw

**Affiliations:** ^1^Department of Animal Science, Agrifood Research and Technology Centre of Aragon (CITA), Zaragoza, Spain; ^2^AgriFood Institute of Aragon – IA2 (CITA-University of Zaragoza), Zaragoza, Spain; ^3^AbacusBio International Limited, Roslin Innovation Centre, Edinburgh, United Kingdom; ^4^Department of Agriculture, Food and Nutritional Science, University of Alberta, Edmonton, AB, Canada; ^5^The Roslin Institute, Royal (Dick) School of Veterinary Sciences, University of Edinburgh, Edinburgh, United Kingdom

**Keywords:** livestock biotechnology, CRISPR, willingness to pay (WTP), added benefits, genetic modification (GM)

## Abstract

Novel gene-editing (GE) technologies provide promising opportunities to increase livestock productivity and to tackle several global livestock production sustainability and food security challenges. However, these technologies, as with previous genetic modification technologies in food production, are very likely to generate social controversy and opposition toward their use in the meat industry. Here, we explored public attitudes and consumption predisposition toward gene-edited meat products and their potential added benefits to livestock farming. Our results show that societal perception currently comes as a package, where the use of gene-editing technology acts as an extrinsic cue of meat products quality, and is used to make a range of inferences about all quality facets at once. Although consumers with anti-GE attitudinal positions generally were not sensitive to price discounts or added benefits, added benefits increased the consumption predisposition of most moderate and pro-GE consumers, where benefits related to animal welfare had larger effects than those relating to the environment or human health issues.

## Implications

We investigated consumer's attitudes and consumption predisposition toward gene-edited meat products and the potential effect of added benefits to consumption predisposition. We found that people's attitudes are formed toward all genetic engineering technologies without differentiating among them. According to our results, the inclusion of gene-edited meat in the food system will likely face societal opposition, and price discounts would not be an effective strategy to modify consumption predisposition. However, the use of gene-editing technology to reduce the negative impacts of livestock production can influence positively public opinion on the use of the technology in meat production.

## Introduction

Novel gene-editing (GE) technologies offer new opportunities to increase agricultural productivity in the context of a growing human population and to tackle several global agricultural sustainability and food security challenges. These opportunities are particularly promising in livestock industries where the application of other genetic modification (GM) techniques has been relatively limited in scope and scale due to technical and social reasons (1, 2). Unlike previous GM techniques, current development of GE technology is already providing ground-breaking capabilities in livestock industries. Researchers have already generated tools to reduce environmental impacts from livestock production [e.g., increased productivity that leads to reduced environmental impact per unit of output; (3)], improve animal welfare [e.g., increase resistance to foot-and-mouth disease in pigs; (4), or animal dehorning; (5)], reduce risks for human health [e.g., elimination of allergens in eggs; (6)], and improve meat production composition and quality (7). As a relatively new technology, the potential of GE to further provide tools to tackle global livestock industry challenges is large. For example, there are already promising GE solutions to increase resistance to two diseases with a significant economic impact: tuberculosis in cows and porcine reproductive and respiratory syndrome in swine (7–9). To unlock the full potential of this technology would significantly improve the livestock industry's capacity to tackle important challenges of livestock production, in the context of a growing human population and increasing societal demands for environmental, animal welfare, human health and food quality improvements.

Some of these societal demands have and will very likely continue to generate controversy and opposition toward the application of GM and GE technologies in the food industry that go beyond the economic and technical issues (10). These controversies will very likely challenge the full deployment of GE technology in livestock industries, despite its promising potential benefits, as they have done before. Previous GM techniques, and especially transgenic technologies applied to plants (11, 12), faced strong opposition, especially in Europe, which, although this has weakened over time, is still significant (13, 14). Society's perceived risk of genetically modified food relates to unknown or unintended impact of human health, animal welfare and environment (15). Bartkowski and Baum (16) singled out three main factors driving societal concerns toward transgenic technologies, which are usually extrapolated by consumers toward all GM techniques: (a) the lack of precision in GM techniques which leads to doubts about undesirable side-effects, (b) the introduction of foreign DNA to the target species from other species or another variety of the same species (trans- and cis- genic, respectively), and (c) that GM technology has been developed and sold by multinational companies and used mainly in intensive crops and is oriented to the use of herbicides. Unlike traditional GM, GE technology does not introduce foreign DNA but, rather, allows the genome to be edited to exhibit desirable traits naturally expressed in other animals of the same (or closely related) species (17). This key difference removes the foundation of the three above-mentioned social concerns.

Literature relating to differential attitudes toward GM and GE technologies are often contradictory, and therefore need further investigation. Some authors have found in foods from plant origin (i.e., rice) that consumers valued gene-edited (i.e., CRISPR) and genetically modified food similarly, and significantly less than conventional food (18). However, other studies show that consumers are able to differentiate between GM technologies and have different attitudes toward them (19–21).

Attitudes toward traditional GM technologies in food production have been widely studied and found to be variable across time and cultures and influenced by several factors [e.g., (22)]. Consumer acceptance of genetically modified food is largely determined by perceived risk and perceived benefits (23). Novel foods in general, and genetically modified products in particular, are generally more acceptable if they provide tangible benefits for the consumer (23, 24). Knowledge and perceived knowledge on GM technologies are generally a key attitudinal driver, possibly by modulating the perceived risk of using the technologies (22). In this sense, Fernbach et al. (25) found that those people with the most negative view toward GM technologies are generally the least informed, though they believe themselves to be well informed. It has long been known that attitudes toward new technologies and GM use in food production vary in relation to the organism involved (animals, plants, microorganism) (23). Therefore, it is highly likely that societal attitudes toward GE food products of animal origin differ to those toward foods of plants origin. This is possibly because use of GE technology in livestock raises ethical issues that do not apply to crops, such as animal integrity and animal welfare, among others (26–28).

Given this social context, it has been argued that, like traditional genetically modified foods, the largest barrier to a widespread use of GE in the food system is not technical, but is in gaining wide-spread public acceptance and understanding (18). Therefore, in order to maximize the potential positive impact of GE technology in livestock production, it is important to understand public attitudes toward gene-edited meat products. However, there are only a few studies that have analyzed social perceptions of GE in livestock and these focus on very specific uses of the technology [i.e., Polled cattle, (29); GE alternative to castration in pigs, (30)]. This study takes a broader approach which complements the specific findings of the above two studies and other studies focusing on gene-edited plant-origin foods [i.e., (18, 31)]. As such, it aims to enhance the understanding of societal attitudes toward meat products from gene-edited livestock in general, and in relation to potential added benefits to livestock farming. Firstly, we assessed societal attitudes toward gene-edited meat products in the context of wider attitudes toward genetically modified food. Secondly, we analyzed consumption preferences based on consumer willingness to pay (WTP) for gene-edited meat products and how this WTP is affected by attitudes and by product benefits related to key societal concerns about to livestock production. Socioeconomic drivers and real and perceived knowledge of both attitudes and consumption preferences were considered. Ultimately, this study provides information to better understand the societal barriers to the adoption and uptake of GE solutions for global food production challenges.

## Materials and Methods

### Questionnaire Design and Survey Implementation

We developed a questionnaire that consisted of four sections: respondent profile, real and perceived knowledge of GE and GM technologies in food production, attitude toward GE and GM technologies in food production, and WTP for GE meat compared to standard meat. A compromise had to be reached between thoroughness, simplicity, and length. The questionnaire was anonymous, to guarantee a higher level of participation and honesty. Personal data were not required, and there was no financial compensation. Participants were clearly informed of the aim of the study and gave implicit consent for the use of their supplied information in the research according to European regulations. The questionnaire was distributed through an online survey developed using Online Survey platform (https://www.onlinesurveys.ac.uk/). United Kingdom (UK) citizens (*n* = 848) were recruited via Paid Facebook advertising (for 8 days) and the social media accounts of the Roslin Institute and the University of Edinburgh. This study was conducted according to the Declaration of Helsinki for studies on human subjects. The questionnaire was approved by the University of Edinburgh Human Ethical Research Committee (HERC).

The respondent profile section of the questionnaire included questions on gender, age, highest level of education achieved, living environment (either rural or urban), being vegetarian or not, and relationship with farming activity (either being a farmer, having a close family member being a farmer, or no relation). To evaluate real and perceived knowledge of GE technology, respondents were first asked how much they know about GE technology or how it can be used in food production. Possible answers were: “nothing,” “a little,” or “a lot.” Then, those who claimed to know either “a little” or “a lot” were asked a follow up question, where they had to choose the definition of GE technology from three options, only one of which was correct. Respondents were given the following five options:

1. “Taking selected genes from one species of animal or plant, and inserting those genes into a different species of animal or plant” (incorrect)
2. “Taking selected genes from an animal or plant, and inserting those genes into another animal or plant of the same species” (correct)
3. “Altering the DNA of an animal or plant using chemicals or targeted radiation to affect selected genes” (incorrect)
4. “None of the above” (incorrect)
5. “Not sure”

After this section, respondents were provided with the following succinct description of GM and GE technology, in order to ensure that they understood the difference between the technologies:

- “Transgenic food: A plant or animal which has had a useful gene transferred from a different species. For example, a cow with a gene transferred from a fish.”
- “Gene-edited food: A plant or animal which has had some of its genes deleted or replaced by genes from another plant or animal of the same species. For example, replacing a gene in a large cow with a gene from a small cow.”

The section on attitudes used Likert-type questions using a 7-point scale ranging from “Strongly disagree” to “Strongly agree”, with the midpoint being “Neutral.” Respondents were asked to state their level of agreement with six statements related to general attitudes toward GM and GE and specific attitude toward ethical, human-health, and environmental aspects of GE, and to the difference between using GE in animals and plants.

Finally, the questionnaire evaluated respondents' WTP for gene-edited meat products compared to “normal” meat. In this exercise, respondents were asked to select which product they would be more likely to purchase (there is an option for no preference) between “normal” chicken breast at a constant price of £6/ kg and gene-edited chicken breast at variable price levels in an iterative process or “bidding game”. We used chicken breast because poultry it is the most widely consumer meat in the UK (32). The bidding game started with both products (i.e., gene-edited and “normal”) at the same price of £6/kg. If respondent chose gene-edited meat or had “no preference” then the bidding exercise ended. If respondent chose “normal” meat, then the question was repeated again with the gene-edited meat at £5/kg (i.e., £1/kg cheaper than “normal” meat). Questions continued until gene-edited meat was priced at £2/kg (i.e., £4/kg cheaper than “normal” meat). If in that final question respondent still chose “normal” meat, they were considered to not consume GE meat under any price scenario.

This exercise was repeated for gene-edited meat with added benefits to evaluate how purchasing behavior change when improvements in animal welfare, environmental impact, and human health are achieved using GE technologies. Specifically, the following three added-benefit scenarios were tested:

1. Added environmental benefits through breeding chickens that have a lower carbon footprint than non-gene-edited chickens.
2. Added human health benefits through breeding of chickens that produce higher levels of Omega 3 than non-gene-edited chickens.
3. Added animal welfare benefits through breeding chickens that are more resistant to certain diseases than non-gene-edited chickens.

In each scenario, the bidding game started with the gene-edited product at £7/kg, this is £1/kg more expensive than the “normal” product option.

### Data Analysis

We used factor and cluster analyses to explore the relationships between attitudes toward different aspects of GE technologies (i.e., ethical, environmental and animal welfare) and to determine if attitudinal groups of individuals could be found. Firstly, we implemented exploratory factor analysis to identify the latent relational structure of the attitudinal aspects explored in the Likert-type questions. We used the “psych” and “GPArotation” packages of R software. The number of factors to select was determined using Horn's parallel analysis (33). We applied an Oblimin rotation and ordinary least squared factoring, which does not assume a multivariate normal distribution. Secondly, we used the root mean square of residuals (RMSR) and the Tucker-Lewis Index (TLI) to validate the factor model. Finally, we implemented k-means cluster analysis on the resulting factors to distinguish attitudinal groups across the sample. The number of clusters was determined by the partition with the highest loss of inertia (within cluster sum of squares). Differences in attitudinal group profiles were evaluated using ANOVA test and Bonferroni pairwise *t*-test for quantitative normally distributed variables and Pearson's chi-squared test for categorical variables.

We analyzed the WTP bidding-game results by comparing the proportion of respondents that prefer the gene-edited product over the standard one, at different price discounts in the different added-benefits scenarios. In addition, differences between attitudinal groups were determined according to their average WTP for gene-edited meat with added-benefits. Differences between groups were evaluated using the non-parametric Wilcoxon signed-rank test. Respondents who would not consume gene-edited meat at any price were not included in the WTP calculation.

## Results

### Attitudes Toward GE Use in Food Production

We found that the latent relational structure of the attitudinal aspects was best described by just one factor. RMSR was 0.05 (should be close to 0) and TLI was 0.987 (should be above 0.9) showing the adequacy of the result. This single factor comprises attitudes toward GM and GE and all human-health, environment, and animal welfare components of GE (Table 1). We call this factor the “Attitude toward GE & GM factor” herein. The statement relating to differential treatment of animals and plants regarding GE is not part of this factor, meaning that this particular attitude is independent of respondents' attitude toward GE and GM.

**Table 1:** Composition of the gene-editing (GE) attitudinal factor.

**Attitudinal statement**	**Factor 1**	**h2**
I have a positive perception toward genetically modified foods	0.90	0.82
I would be comfortable eating food produced using GE technology	0.95	0.90
GE in food production is ethical	0.92	0.85
GE in food production is safe for human health	0.95	0.90
GE in food production is safe for the environment	0.93	0.87
GE in animals and plants used for food production should be treated differently	−0.08	0.01
Proportion of variance explained	0.72	

*Standardized loading of attitudinal statements*.

We ran the cluster analysis on two variables: the Attitude toward GE & GM factor and the (typified) variable corresponding to the attitudinal statement related to differential treatment of plants and animals (last statement in Table 1). The cluster analysis determined the existence of the following four groups of respondents (Table 2):

1. Anti-GE, Kingdom indifferent (18.9% of respondents): Respondents in this group had a very negative attitude toward GE and GM in food production, and consistently consider that animal and plant kingdoms should be treated in the same way when using GE for food production. Since this group made no distinction between animal and plant kingdoms, we called it Kingdom indifferent.
2. Anti-GE, Kingdom different (27.6%): Respondents in this group have a negative attitude toward GE and GM, but contrary to the previous group, they strongly believe that plant and animal kingdoms should be treated differently for GE in food production.
3. Moderate (42.1%): It is the largest group of respondents in the sample. They have neutral or slightly positive attitudes toward GE and GM in food production and consider that animals and plants should be treated differently.
4. Pro-GE (11.4%): This is the smallest attitudinal group in the sample. This group has very positive attitudes toward GE and GM, and strongly considers that plants and animals should not be treated differently.

**Table 2:** Description of attitudinal groups according to the variables used in the cluster analysis.

**Attitudinal group**	***n***	**^a^Attitude toward gene-editing and genetic modification (factor)**	**Attitude toward differential treatment of animals and plants in gene-editing (typified variable)**
Anti-gene-editing, Kingdom indifferent	160	−0.98^A^ ± 0.37	−1.35^A^ ± 0.34
Anti-gene-editing , Kingdom different	234	−0.81^B^ ± 0.39	0.87^D^ ± 0.54
Moderate	357	0.57^C^ ± 0.5	0.29^C^ ± 0.47
Pro gene-editing	97	1.31^D^ ± 0.39	−1.05^B^ ± 0.4
Total	848	0.0 ± 1.0	0.0 ± 1.0

a *Negative values refer to negative attitudes toward gene-editing and genetic modification and that animals and plants should be treated in the same way. All consumer groups showed significant differences for the attitudinal factors according to ANOVA tests (P > 0.001)*.

A−D *Different letters indicate statistically significant differences between consumer groups according to Bonferroni pairwise t-test (P < 0.001)*.

**Figure 1:**
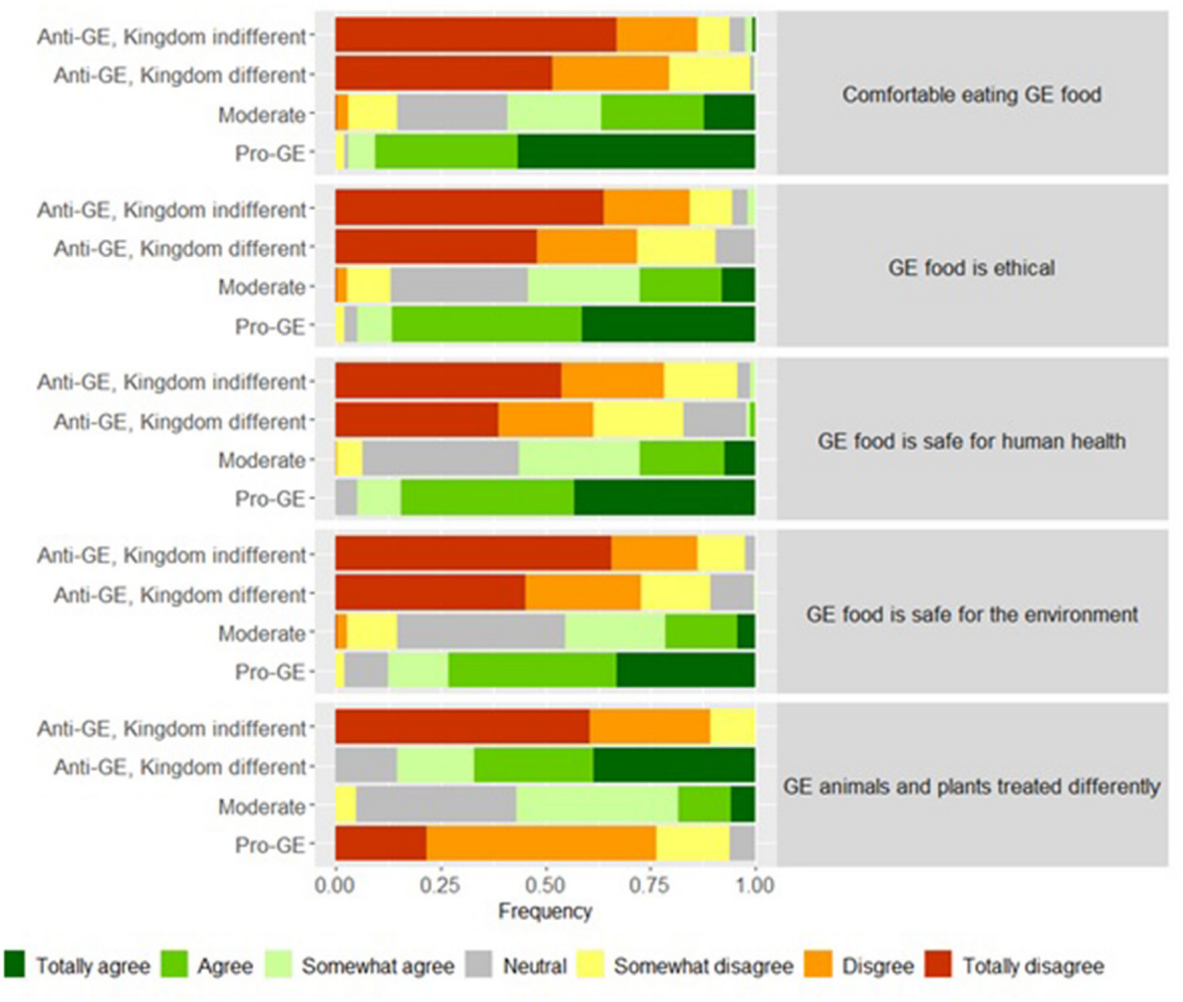
Attitude toward different aspect related to gene-editing (GE) in food production across attitudinal groups.

A more detailed description of the distribution of the attitudinal positions regarding GE in each group is presented in Figure 1.

### Socioeconomic Drivers of Attitudes

We found that attitudinal groups have different demographic profiles (Table 3). Groups with a more positive attitude toward GE and GM are associated with youth, being male, consuming meat, and living in an urban environment. There were no clear differences between attitudinal groups regarding education level, employment situation, or relationship with farming (*P* > 0.05).

**Table 3:** Description of attitudinal groups; age, and proportion of females, vegetarians, and urban dwellers.

**Attitudinal group**	***n***	**Mean age**	**Females (%)**	**Vegetarians (%)**	**Urban (%)**
Anti-gene-editing, Kingdom indifferent	160	57.4^A^ ± 12.2	74.5%	21.9%	49.4%
Anti-gene-editing, Kingdom different	234	56.9^A^ ± 14.1	82.5%	28.2%	56.0%
Moderate	357	50.5^B^ ± 18.2	72.0%	14.0%	63.9%
Pro- gene-editing	97	43.1^C^ ± 17.4	49.5%	9.3%	69.1%
Total	848	47.6	68%	19%	61%
ANOVA test *p*-value		*P* < 0.001			
Chi^2^ *p*-value			*P* < 0.001	*P* < 0.001	*P* < 0.001

A−D * Different letters indicate statistically significant differences between attitudinal groups, calculated according to Bonferroni pairwise t-test (P < 0.001)*.

Respondents that claimed to have no knowledge of GE had a less favorable attitudes toward GE & GM than respondents that declared some knowledge (either “a little” or “a lot”; Table 4), however, there was no (statistical) differences in attitude between those who believed to know “a lot” about GE and those who claimed to know “a little”. On the contrary, real knowledge about GE technology had no influence on attitudes. There were no statistical differences in the weight of the Attitude toward GE & GM factor between respondents who got the correct definition of GE technology, respondents who got it wrong, and respondents who were unsure about it. Furthermore, 57.1% of the people that claimed to know “a little” about GE technology and 78.8% of the people that claimed to know “a lot” were not able to select its correct definition. Note that real knowledge was only determined for respondents claiming to have some knowledge of GE, either “a little” or “a lot”.

**Table 4:** Relation between attitude and perceived and real knowledge toward gene-editing technology.

	**Number of respondents (%)**	**Attitude toward gene-editing and genetic modification factor (average and SD)**
**Perceived knowledge of gene-editing technology**		
None	287 (28.5%)	−0.19^A^ ± 0.77
A little	439 (51.4%)	0.00^B^ ± 0.94
A lot	122 (20.1%)	0.33^B^ ± 1.19
^a^ **Real knowledge; respondents guessing right the true definition of gene-editing technology**		
Correct	157 (27.8%)	0.14 ± 0.96
Incorrect	345 (61.1%)	0.09 ± 1.04
Not sure	63 (11%)	−0.17 ± 0.92

a *Real knowledge was only determined for respondent declaring to have some knowledge on gene-editing, either “a little” or “a lot”*.

A−D *Different letters indicate statistically significant differences between groups by perceived knowledge, calculated according to Bonferroni pairwise t-test (P < 0.001)*.

### Willingness to Pay for Gene-Edited Meat Products

**Figure 2:**
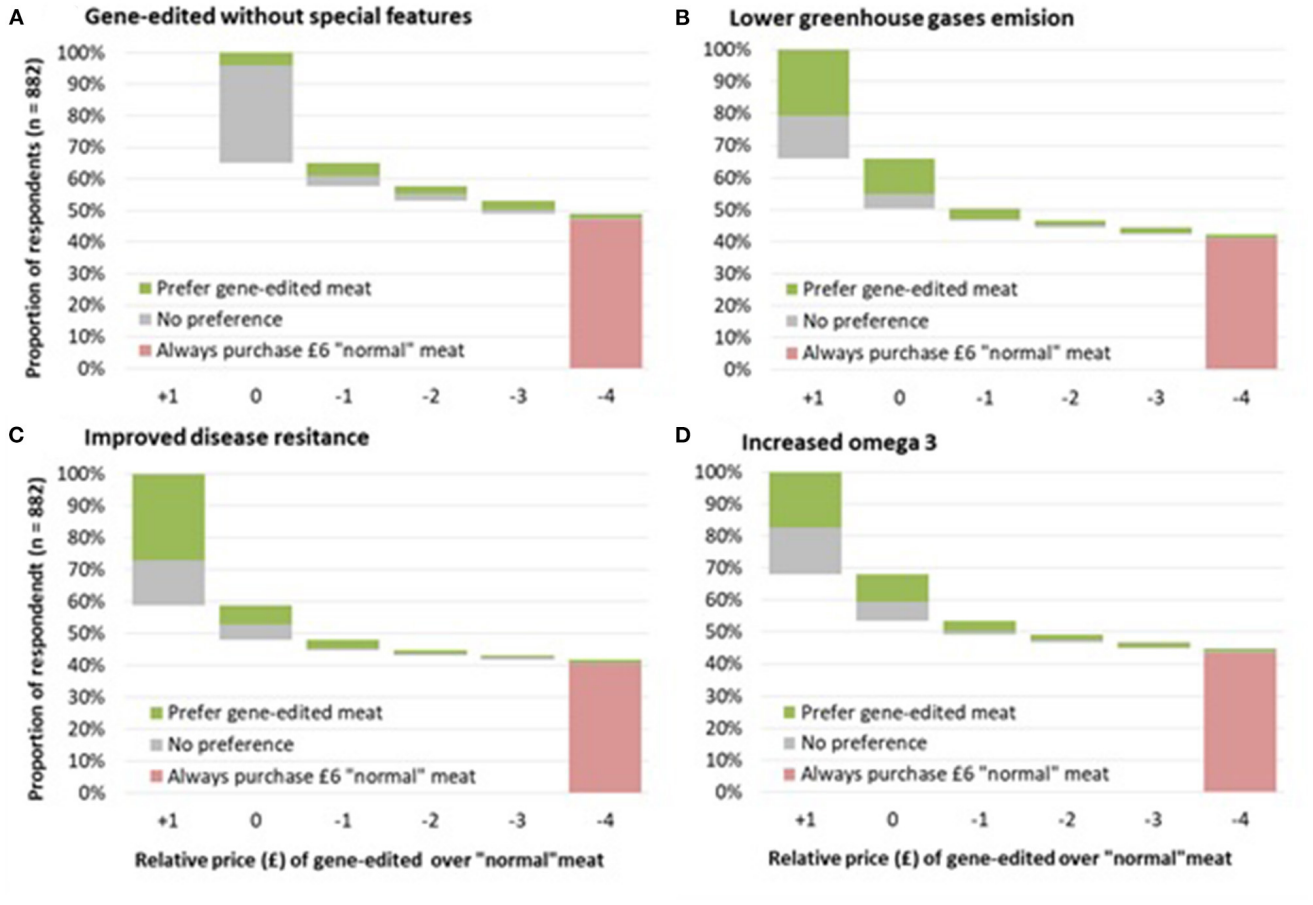
Willingness to pay for gene-edited meat (chicken breast) by its relative price compared to normal meat (£6/kg.).

Figure 2 and Table 5 illustrate the results of the WTP exercise. Almost half (47.1%) of respondents stated that they would always choose “normal” meat instead of gene-edited meat regardless of the price discount. Adding benefits to gene-edited meat slightly changed this proportion; 40% would always choose normal meat instead of GE meat with improved animal welfare, 41% for lower environmental impacts, and 43.6% for increased human health benefits. On the other side, at equal prices 35% of respondents either prefer gene edited meat (without special features) or have no preference between gene-edited meat or “normal” meat. Finally, 17.9% of the respondents chose gene-edited meat when price discounts were offered. When respondents were asked to consider gene-edited meat in the context of additional benefits associated with GE technology, a large proportion of respondent would pay a premium of £1/kg (41.3% for improved animal welfare, 34.0% for lower environmental impact, and 31.8% for increased human health benefits).

**Table 5:** Willingness to pay for gene-edited meat with different beneficial features across attitudinal groups.

**^1^Willingness to pay (£) for gene-edited meat products**	**Attitudinal group**				
	**Anti-gene-editing, Kingdom indifferent (*n* = 125)**	**Anti-gene-editing, Kingdom different (*n* = 168)**	**Moderate (*n* = 307)**	**Pro-gene-editing (*n* = 88)**	**All (*n* = 688)**
Without special features	−2.10^a^^b^ ± 1.6	−2.05^a^ ± 1.28	−0.72^b^ ± 1.13	−0.14^c^ ± 0.46	−0.69 ± 1.14
From animal with increased disease resistance	−0.87^a^ ± 2.17	−0.19^a^^b^ ± 1.63	0.49^a^^b^ ± 0.99	0.73^b^ ± 0.6	0.43 ± 1.11
From animal with lowered GHG emission	−1.08^a^^b^ ± 1.94	−0.89^a^ ± 1.81	0.35^b^^c^ ± 1.03	0.59^c^ ± 0.62	0.25 ± 1.18
With increased Omega3 content	−1.0^a^^b^^c^ ± 2.16	−0.72^a^ ± 1.69	0.27^b^ ± 1.1	0.54^c^ ± 0.69	0.22 ± 1.19

1 *Average and SD willingness to pay among those respondents willing to consume gene-edited meat. Negative values refer to discount required by consumers in order to purchase*.

a−c *Different letters indicate statistically significant differences between consumer groups, calculated according to Pairwise t-test variance (P < 0.05)*.

**Figure 3:**
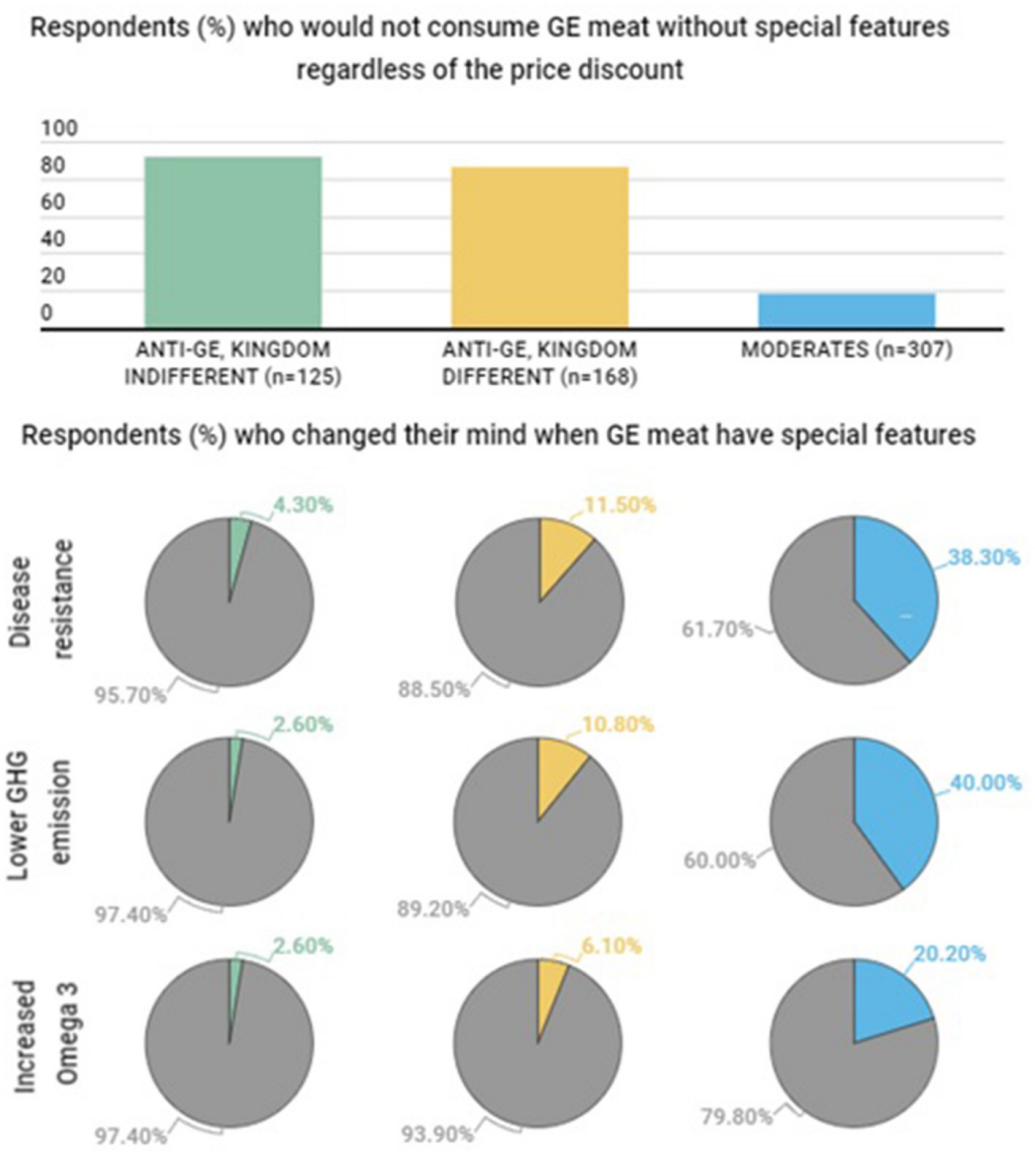
Influence of added benefits in gene-edited (GE) meat consumption across attitudinal groups.

Attitudinal groups clearly differentiated in their WTP for gene-edited meat and consumption predisposition (Table 5). On the one hand, “Anti-GE” groups had a lower WTP for gene-edited meat than the “Moderate” and the “Pro-GE” groups. Average WTP is negative in all groups but in “Pro-GE”, (which is very close to 0), meaning that price discounts were required for them to purchase the gene-edited meat. Furthermore, most people in “Anti-GE” groups (91.5 and 96.5% in “Kingdom indifferent” and “Kingdom different” groups, respectively) stated that they would not consume gene-edited meat regardless of the price discount and the associated benefits (Figure 3).

### Influence of Additional Benefits on WTP for Gene-Edited Meat Products

When considering gene-edited meat with additional benefits, WTP increased in all attitudinal groups; “Anti-GE” groups still showed a negative WTP, but both “Moderate” and “Pro-GE” groups showed a positive WTP (Table 5). Across all attitudinal groups, WTP was highest when benefits were associated with improving animal welfare (increasing animal disease resistance) and lowest when benefits were associated with human health (increased Omega 3 levels).

Finally, we found that some respondents that previously stated they would not eat standard gene-edited meat regardless of the price discount changed their mind when benefits were introduced (Figure 3). The proportion of respondents that changed their mind varied across attitudinal groups. Close to half of the “Moderate” group (40%) changed their mind, but only a small proportion of “Anti–GE, Kingdom different” (11.5%) and “Anti–GE, Kingdom indifferent” (4.3%) groups would do so. Again, across all attitudinal groups, benefits associated with animal welfare and lowering GHG emissions where more important than benefits for human health.

## Discussion

Societal opposition to GM use for food production has limited its adoption in agriculture, especially in European countries. Although nowadays there is less societal debate about GM technology than a decade ago, a large part of the society is still concerned about it use in the food industry [e.g., (13, 14)]. This historic debate focused on GM crops because its application to livestock production was very limited. Because new GE technology developments have been successfully applied in livestock [e.g., (4–7)], there is a renewed interest in analyzing the people's specific attitudes toward its use in meat production. However, only a few studies have analyzed societal attitudes toward specific uses of GE technology in livestock production. To our knowledge, this study is the first to analyse general consumers' attitudes toward GE technology in livestock production and WTP for gene-edited meat products with and without potential animal welfare, environmental, and human health benefits.

The results of our investigation add to a growing body of research which suggests that society will view gene-edited foods similarly to how they view genetically modified foods (18, 22), which implies that inclusion in the food system would be controversial. We found similar attitudinal groups and drivers of attitudes and consumption predisposition (i.e., age, gender, place of living and perceived knowledge) than previous studies. Our results also show that attitudes are likely to be positively affected by added benefits. Finally, unlike the use of GE in crops, gene-edited meat raises issues related to animal welfare, which affects both the intrinsic components of attitudes toward GE, and people's evaluation of potential benefits of this technology. These issues are discussed in detail below.

### Attitudinal Dimensions

According to our study, attitudes toward gene-edited meat products are built on two independent attitudinal dimensions: the attitude toward GM and GE technologies in food production, and the attitude toward the differential treatment of animals and plants. Attitudes (either positive or negative) toward the use of GE technology for food production are consistently created toward the “whole package” of GE and all its facets related to ethical aspects, human health, and environmental issues, without distinguishing between them, along with genetically modified foods in general. This result shows that GE possibly functions as an extrinsic cue (i.e., signal) of food product quality (34), similarly to how meat origin, and animal feed or production system can signal food product quality (35). In this sense, GE would work as a consumer heuristic that backs up a story of the production process, which is used by people to make a whole range of inferences about product quality, leaving no space for nuance.

### Attitudinal Groups

The combination of both attitudinal dimensions in a factor analysis allowed us to identify four attitudinal groups of people; two anti-GE, one moderate, and one pro-GE. The opinion on the differential treatment of animals and plants was key to segmenting attitudinal positions, with the most extremely pro-GE attitudinal groups not differentiating between animals and plants under any circumstance. The two anti-GE groups differentiated in their position toward treating animal and plants differently. The existence of anti (“pessimistic”), moderates (“undecided”), and pro (“optimistic”) groups have been consistently found by several authors when studying people perception on genetically modified food [e.g., (22)]. Unlike most of these studies which found that the “optimistic” group was usually rather large, our results show that when it comes to GE meat products, negative and moderate attitudinal positions dominate public opinion, with only a small proportion of respondent having pro-GE attitudes beyond doubt. The greater reluctance to use GE in livestock compared to using it in plants is very likely related to the great public concern for farm animal welfare (36, 37). Note that most respondents in our study consider that plant and animals are not the same and therefore should be treated differently in regard with GE.

### Attitudinal Drivers

Our study shows that youth and gender (i.e., males), and to a lesser extent place of living (i.e., urban), and non-vegetarianism, influenced positively attitudes toward GE use in meat production. These results are in line with previous studies which found that age and gender generally influence attitudes toward using GM technology in food production [e.g., (22, 38, 39)] and livestock welfare issues [e.g., (40–42)]. Similarly, urban inhabitants are usually found to have a more positive attitude toward GM use in food production [e.g., (43, 44)].

Perceived knowledge on genetically modified food has been widely studied, and is usually separated between real (tested) knowledge and perceived (self-assessed) knowledge. In accordance with House et al. (45), we found that the lower the perceived knowledge of the respondent on GE technology the more negative the attitude toward it (and the lower WTP for GE meat products). Low knowledge increases risk perception, which has been proven to be strongly related to GM acceptability (22, 46). Contrary to other authors [e.g., (22, 45, 47)], we found no relationship between real knowledge and attitudes or WTP, however, these differences may be due to differences in the way “knowledge” is measured across studies (45).

### Consumption Predisposition, WTP and Perceived Benefits

The WTP exercise showed that respondent predisposition to consume gene-edited meat products is negative (i.e., price discounts are generally required) and is not influenced by further price discounts in large share of the respondents. Almost half of respondents would not consume gene-edited meat products regardless of the price, while around one third of the sample would have no problem consuming it. This means that only a small proportion of respondents (around 15%) were shown to be sensitive to price discounts. As expected, WTP from gene-edited meat products is very much related to attitudes toward it, with Anti-GE groups showing a much lower (negative) average WTP and a lower sensitivity to price discounts than Moderate and Pro-GE groups.

It is widely known that the use of GM to get added benefits increases consumers' acceptability and WTP for genetically modified products [e.g., (23, 24, 48)]. This has proved to also hold true for specific examples of GE technology use for increasing animal welfare in livestock production; i.e., polled cattle (29) and alternative to castration in pigs (30). We indeed found, in a more general approach, that added benefits increased WTP for gene-edited meat products and that the effect was larger on benefits related to animal welfare, than to environmental or human health issues. This finding fits with the high relative importance that western society gives to animal rights within livestock production, and the higher importance of animal welfare compared to other livestock challenges [e.g., (29, 38)]. However, our study also shows that the respondent attitudes are affected by added benefits to a limited extent and differently across attitudinal groups. Most of the people who hold Anti-GE positions would not consume gene-edited meat products regardless of the price discount and they would not modify their consumption predisposition when either animal welfare, environmental or human health benefits are added. On the contrary, most Moderates and Pro-GE respondents attitudes are sensitive to added benefits. Actually, Moderates and Pro-GEs people, who on average would require price discounts to consume gene-edited meat products, would on average be willing to pay overprice (0.27–0.73£/Kg depending on the type of benefit) for gene-edited meat with added benefits. Our research suggests that GE technology use in meat production would initially be acceptable to around half of consumers, although most of them would require a price discount or added-benefits to prefer gene-edited meat over normal meat.

### Limitations

This study has a number of limitations that should be acknowledged. Firstly, participants were recruited via online advertising, which, although provides a practical, cost-effective, and efficient way to gather a large and diverse sample, might bias the sampling toward internet users. Given the large usage of internet in UK households (90% of homes, 2020), we do not expect a large bias in this regard. We should also note that our sample might be slightly biased toward anti-GE positions as social groups that showed more negative attitudes toward GE use in meat production (rural, females, and aged) were to some extent overrepresented in the sample compared to overall UK population [(49); Supplementary Table 1]. Therefore, care is required when making inferences of the results of the survey about the whole UK population.

Similarly, our study was focused on the UK so its results cannot be immediately extrapolated to other countries. Previous studies on attitudes toward GM and GE food products across regions and countries [e.g., (18)], generally found similar attitudinal behaviors across western countries, with European citizens being the ones showing the greatest concern. Therefore, the results of our study only apply to UK. However, given the similarity of UK society with other European societies, with regard to attitudes toward GM and GE technology uses in food production, we would not expect results in other European countries to be very different. Extrapolation out of Europe should be done with care.

We should also note that attitudes that consumers express toward food products are not (always) strongly related to purchase behavior. However, this does not necessarily mean that attitude does not affect other behaviors, for example political behavior (34). Therefore, we should not interpret the implications of the results of our study only in terms of its impact people's role as consumers, but also in terms of potential influence on people's role as citizens. Currently, livestock production and meat consumption are important issues politically [e.g., (26, 50)]. Therefore, negative attitudes toward gene-edited meat found in our study might not (only) have a large effect on the future consumption of potential products, but are also likely to have a strong influence on public opinion and in turn in policy and regulatory decisions.

Finally, gene editing technology is still a largely unknown among the general public, which presumably will change as the technology develops and its adoption in the farming sector spreads. Since people's attitudes are largely influenced by their knowledge, as discussed above, it is possible that that the attitudes reflected in this study change soon, particularly given the interest of GE technology developers in making society distinguish between this technology and traditional GM.

### Implications for GE Technologies Development

As GE technologies continue to advance, society must make decisions about their role in the food system. There are two clear messages emerging from our study. First, that perception of these technologies currently comes as a package; individuals start from either a supportive or a concerned stance for all genetic engineering technology. Our results add to a growing body of research which suggests that society will view gene-edited foods similarly to how they view genetically modified foods. The second conclusion is that there is a need for continued dialogue to provide the information that individuals seek. There remains an opportunity to differentiate people's perceptions between GE and GM, while our data strongly supports the need to communicate the benefits the technology offers to society. If there are real differences in the application and benefits of the different genetic engineering technologies, then these need to be better articulated to enable society to develop informed opinions. Consumer decisions on whether or not to buy GE food is not fixed, and changes in opinion remain possible. Changes will be reliant on clear, transparent dialogue around the benefits that the technology can deliver to society. The ability to appropriately communicate the improvements of GE technology over previous GM techniques on issues of high importance to society, like meat quality, environmental impact and animal welfare, will likely shape the evolution of public attitudes toward it use in meat production, and in turn affect how the sector develops. In parallel, fair societal concerns around the ethics of artificially modifying animals' genomes remain, and these will continue to influence this dialogue. All actors have a role to play in the dialogue, from transparent representation by industry, to informed decision making by stakeholders, with trusted information sources likely to reside in recognized academic institutions. More research is needed to investigate the relationship between attitudes toward GE technologies and different messaging and communication strategies, and how consumers respond to labels highlighting different information or positive benefits associated with GE technology.

## Data Availability Statement

The raw data supporting the conclusions of this article will be made available by the authors, without undue reservation.

## Author Contributions

DM-C: methodology, formal analysis, writing—original draft, writing—review and editing, and visualization. TB: conceptualization, methodology, investigation, resources, writing—review and editing, supervision, and project administration. JC: methodology, formal analysis, investigation, writing—review and editing, and visualization. TK: conceptualization, methodology, formal analysis, investigation, writing—review and editing, and visualization. GR: writing—review and editing. CW: conceptualization, resources, writing—review and editing, supervision, and funding acquisition. All authors contributed to the article and approved the submitted version.

## Funding

The authors would like to thank the Biotechnology and Biological Sciences Research Council (BBSRC) for supporting this research, which was funded by a grant for the Roslin Institute, from BBSRC's Industrial Strategy Challenge Fund: Transforming Food Production initiative. CW was supported by the BBSRC through Institute Strategic Programme grants BB/P013732/1 and BB/P013759/1.

## Conflict of Interest

TB, JC, and TK were employed by AbacusBio International Limited. The remaining authors declare that the research was conducted in the absence of any commercial or financial relationships that could be construed as a potential conflict of interest.

## Publisher's Note

All claims expressed in this article are solely those of the authors and do not necessarily represent those of their affiliated organizations, or those of the publisher, the editors and the reviewers. Any product that may be evaluated in this article, or claim that may be made by its manufacturer, is not guaranteed or endorsed by the publisher.
