# Supplementary material for: Gene-Edited Meat: Disentangling Consumers' Attitudes and Potential Purchase Behavior
Source: Front Nutr. 2022 Apr 5;9:856491. doi: 10.3389/fnut.2022.856491 (PMC9017286; doi:10.3389/fnut.2022.856491)
Supplement: Supplementary file 1 [file Table_1.docx]

**Supplementary material**

**Supplementary Table S1. Comparison of survey respondent age distribution with the overall UK population**

| Age category | Number of respondents | Percentage | Actual UK age distribution^1,2^ |
| --- | --- | --- | --- |
| <20 | 25 | 2.6% | na |
| 20-29 | 151 | 15.6% | 17.9% |
| 30-39 | 107 | 11.0% | 17.3% |
| 40-49 | 103 | 10.6% | 19.3% |
| 50-59 | 172 | 17.7% | 16.0% |
| 60-69 | 265 | 27.3% | 14.2% |
| 70+ | 148 | 15.2% | 15.3% |
| n total | 971 | 100.0% | 100.0% |
| ^1^ Office for National Statistics (2013), 2011 Census: Population Estimates by five-year age bands, and Household Estimates, for Local Authorities in the United Kingdom | | | |
| ^2^ The <20 age category has been excluded from the actual UK age distribution because the survey was not targeted at people aged younger than 18. | | | |
